# Multi-Mycotoxin Occurrence in Dairy Cattle Feeds from the Gauteng Province of South Africa: A Pilot Study Using UHPLC-QTOF-MS/MS

**DOI:** 10.3390/toxins10070294

**Published:** 2018-07-16

**Authors:** Rumbidzai Changwa, Wilfred Abia, Titus Msagati, Hlengilizwe Nyoni, Khanyisa Ndleve, Patrick Njobeh

**Affiliations:** 1 Department of Biotechnology and Food Technology, Faculty of Science, University of Johannesburg, Doornfontein Campus, P.O. Box 17011, Doornfontein Campus, Gauteng 2028, South Africa; abiawilfred@yahoo.com (W.A.); khanyindleve@gmail.com (K.N.); 2 Nanotechnology and Water Sustainability Research Unit, College of Science, Engineering and Technology, University of South Africa, Roodepoort, Johannesburg 1710, South Africa; nyonih@unisa.ac.za

**Keywords:** mycotoxins, dairy feeds, UHPLC-MS/MS, South Africa

## Abstract

The indispensable nature of toxigenic fungi and mycotoxins in agricultural systems is of worldwide concern, hence the need for surveillance studies to preserve public health. Thirteen dairy farms were surveyed and 40 dairy feeds of varying nature collected and analyzed for mycotoxins. Estimated levels of aflatoxins (AFs), fumonisin B_1_ (FB_1_), ochratoxin A (OTA), citrinin (CIT), zearalenone (ZEN), α-zearalenol (α-ZEL), β-zearalenol (β-ZEL), deoxynivalenol (DON), 3- and 15-acetyl-deoxynivalenol (ADONs), HT-2 toxin (HT-2), and beauvericin (BEA) were established using liquid chromatography-tandem mass spectrometry. Highest frequencies (40/40) were found for AFG_2_ (range: <LOQ—116.1 ppb), α-ZEL (range: 0.98–13.24 ppb), and β-ZEL (range: 0.73–4.71 ppb), followed by AFB_2_ at 37/40 (range: <LOQ—23.88 ppb), BEA at 36/40 (range: <LOQ—55.99 ppb), HT-2 at 35/40 (range: <LOQ—312.95 ppb), and FB_1_ at 34/40 (range: <LOQ—1389.62 ppb). Apart from samples exceeding regulatory limits for total AFs in dairy feeds due to the high amounts of AFG_2_ and AFB_2_, levels of other mycotoxins were regarded as safe for dairy production in South Africa. This is the first-time the natural occurrence of the cold climate HT-2 in South African feeds was documented. Persistent co-occurrence of multiple mycotoxins across samples, however, may elicit synergistic and/or additive effects in hosts, hence raising concerns about their impacts and how such interactions may affect the dairy livestock sector.

## 1. Introduction

Mycotoxins, which are toxic fungal metabolites, remain a serious problem associated with food and farming systems for both human and animals worldwide. Dairy farming is largely affected, with regards to feed contamination and the risks posed not only to productivity but the ability of these organic contaminants to reach the human diet through animal by-products. In South Africa, dairy farming contributes significantly to the national economy with most farms concentrated in the eastern and northern Free State, the KwaZulu-Natal Midlands, Eastern and Western Cape, Gauteng, and southern parts of Mpumalanga [1,2]. However, with animal feeds being an indispensable component along the food chain from farm to fork [3], transmissible hazards such as mycotoxins along the food chain pose a significant threat to human health.

Chronic or acute exposure of animals to mycotoxins via contaminated feeds may result in nephrotoxic, hepatotoxic, carcinogenic, neurotoxic, immunotoxic, oestrogenic, mutagenic or teratogenic effects [4,5,6]. These effects, generally termed mycotoxicosis may serve as risk factors to several other animal and human diseases (e.g., increased susceptibility to cancers) and may even result in death [7]. Apart from these varying health effects in animals, there is also an eminent potential for mycotoxin carry-over from dairy animals to humans via contaminated by-products such as meat and particularly milk. This is the situation especially since significantly high mycotoxin levels, above regulation, have been reported in South African animal feeds [3,8] as well as milk products [9,10] in the past. This is worsened by the fact that most farming communities, particularly those among rural populations are not aware of the apparent risks linked to mycotoxin contamination of feeds.

Thus, while there is a need to understand the perception of farmers on mycotoxin contamination and associated health problems it is also imperative to estimate quantities of various mycotoxins in animal feeds for risk assessment and communication. Therefore, it is important to monitor the levels of multiple mycotoxins in animal feeds regularly to assist in the building of periodic monitoring databases based on analytical data to evaluate country’s risk profile and for effective management. These will safeguard animals, and in turn human health while boosting the economy. While this is performed in the developing countries, it is limited in South Africa as only very few studies [3,8,11] have been reported in the literature on multi-mycotoxin contamination of animal feeds. Additionally, despite the increased interest on mycotoxins in South Africa, few studies have focused on mycotoxin contamination of feeds, particularly dairy cattle feeds. Therefore, it is relevant to provide regional information on the levels of multiple mycotoxins as well as their biotransformation products in dairy feeds from South Africa, with a hope of providing somewhat the basis to explore proper intervention strategies that can be put in place to reduce animal exposure levels. These will safeguard animals and in turn human health, while also boosting the economy and social status of the people of South Africa. The objective of this study was therefore to assess the natural occurrence and levels of several mycotoxins in dairy cattle feeds utilized in a representative population of South African dairy farms in 2015 while evaluating farmer perceptions to better understand mycotoxins contamination in feeds.

## 2. Results

### 2.1. Farmer Perception Survey

Analysis of data retrieved from questionnaires administered to participating farms revealed farmer characteristics indicating that the majority (>80%) had been in the commercial (small and large scale) dairy farming business for over 10 years. Common cattle breeds here included Holstein, Jersey, hybrids of the two, and the not so common Friesian and Ayrshire. Herd size ranged from as little as 20 to >700 lactating cows and milk yield from approximately 300 to 3000 L/day. Animal diets consisted of maize/grass silages, forage, commercial compound feeds, maize, oilcake, and cereal feeds (maize, bran, and brewers grain).

**Figure 1:**
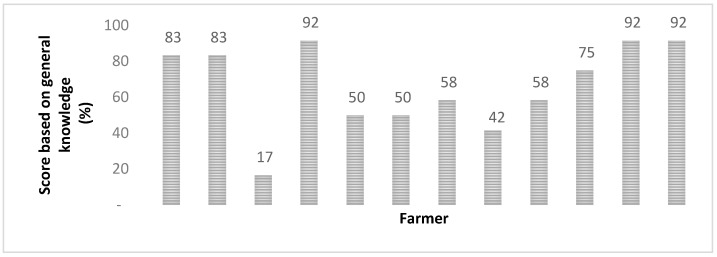
Individual farmer scores on their general mycotoxin knowledge (%).

Regarding the individual farmers’ general knowledge of mycotoxins, causes, and control strategies, data revealed that all respondents had a general knowledge on the topic of mycotoxins and their associated risks with knowledge scores ranged from 17–92% (Figure 1). It is, however, important to note that of all the farms evaluated in Gauteng, none of the dairy cattle showed visible signs of mycotoxicosis.

Here individual farmer knowledge was scored by assessing their responses to questions on general mycotoxin-related topics, with specific regard to where they thought mycotoxins come from, what conditions promote their presence in storage, effects on their animals, legislation, and control strategies implemented. Of the surveyed farmers, 67% showed familiarity with the term ‘mycotoxin’, while only 5/8 of these knew that mycotoxins were in fact produced by fungi. When asked specifically about the effects mycotoxins have on dairy, cattle two-thirds of the farmers stated conditions within reason (death, abortions, illness, diarrhea, liver malfunction and decreases in milk production) while the other third did not know. Of concern, however, only three participating farmers confirmed having them or their management attending feed safety or mycotoxin-related workshops in the past.

Almost all farmers stated one method or another of how they cater for possible mycotoxin outbreaks on their farms. Typical farmer responses illustrated 75% (9/12) of the farmers practicing on-farm methods such as ‘first in first out’ at storage and 50% (6/12) of them further describing what they thought was ensuring adequate storage (sanitation, keeping feed bags for the least time possible, adequate ensilaging techniques and minimizing moisture in stores). Thirty-three % (4/12) of farmers laid emphasis on drying and sorting as post-harvest methods. However, only 2 of the 12 farmers surveyed use mycotoxin binders in their feeds with only one of two further detailing mycotoxin testing as a preventative measure. Overall insights revealed a limited understanding mycotoxin legislation and modern forms of mitigation strategies they may have access to as farmers.

### 2.2. Qualitative Screening Identification

**Figure 2:**
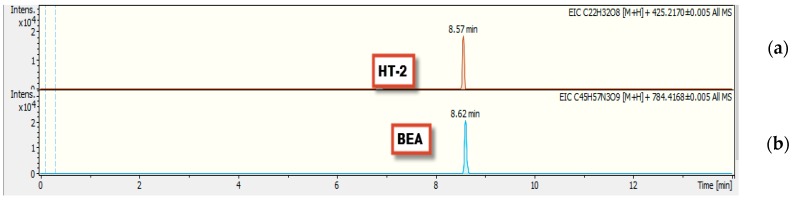
Extracted ion chromatograms (EIC) of high resolution accurate mass LC-MS of mycotoxins [M + H]^+^ in standard mix; (**a**) HT-2 toxin [*m/z* 425.2170] and (**b**) Beauvericin [*m/z* 784.4168]. The extracted ion window was 0.4 min of the exact *m/z*.

**Figure 3:**
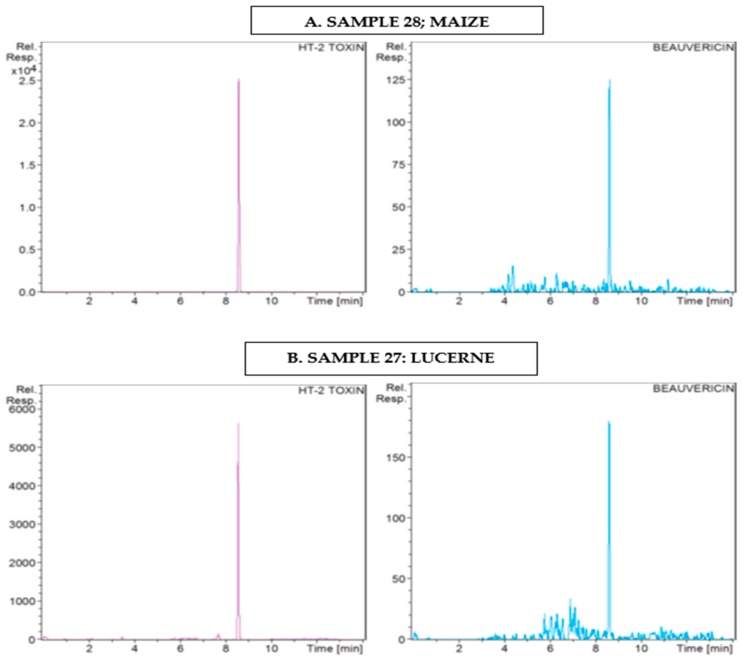
Corresponding EIC of HT-2 Toxin (*R_T_* 8.57) and beauvericin (*R_T_* 8.62) in different sample matrices showing similar retention profiles as reference standards.

An assessment on the frequency of mycotoxins in dairy feedstuff from different farms in the Gauteng province of South Africa revealed the occurrence of 13 out of the 15 mycotoxins studied. Targeted UHPLC-QTOF-MS/MS (ultra-high-performance liquid chromatography coupled with quadrupole time-of-flight tandem mass spectrometry) allowed for the accurate identification of target mycotoxins based on retention profiles, precise molecular masses, and MS spectra at selected MS parameters in positive ionization mode. LC-MS allowed for the metabolite annotation by matching retention times and elution profiles of analytes to that of the calibrate standards from resultant chromatograms as shown in Figure 2 and Figure 3.

**Figure 4:**
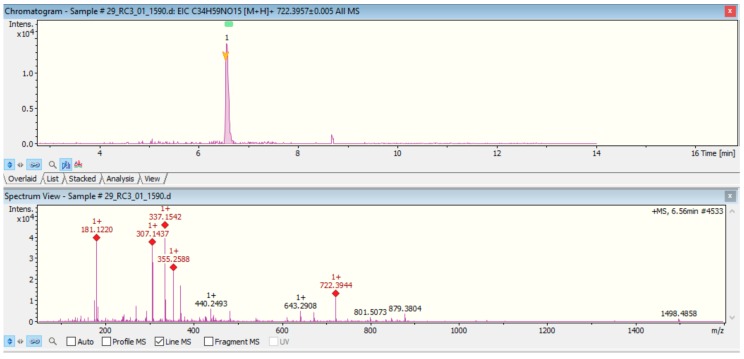
Chromatogram and MS spectra for FB_1_ [*m/z* 722.3957] used in mass and spectra-based searches.

**Figure A2:**
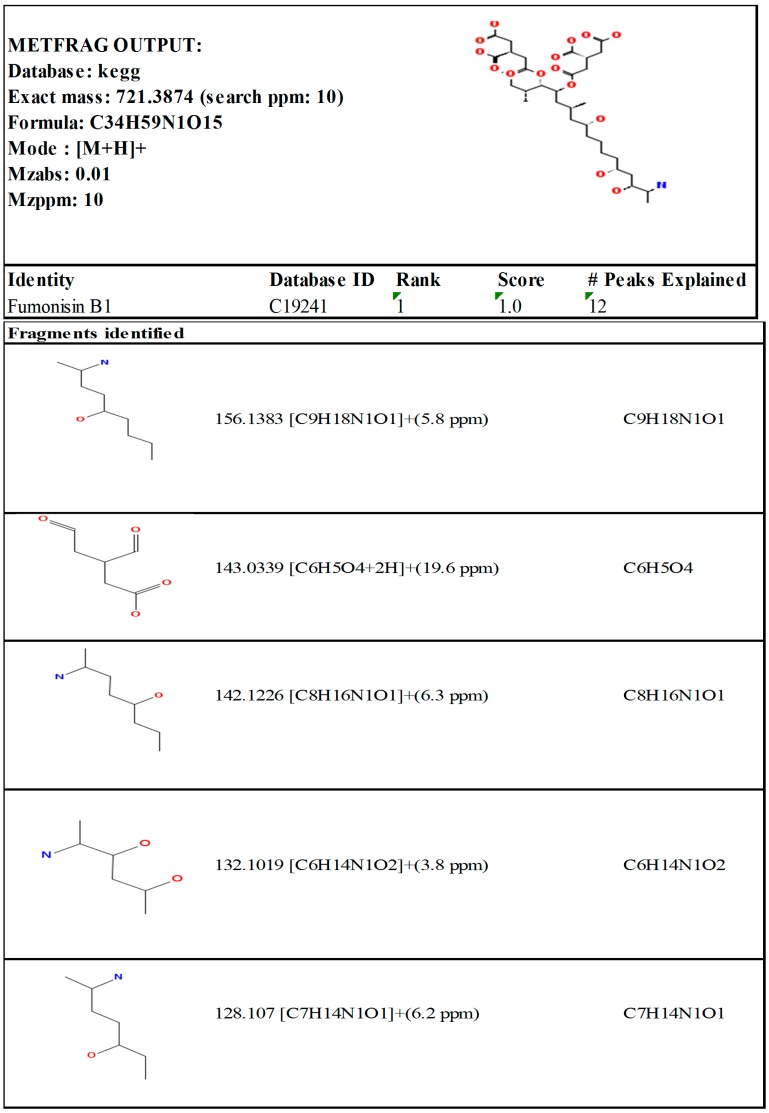

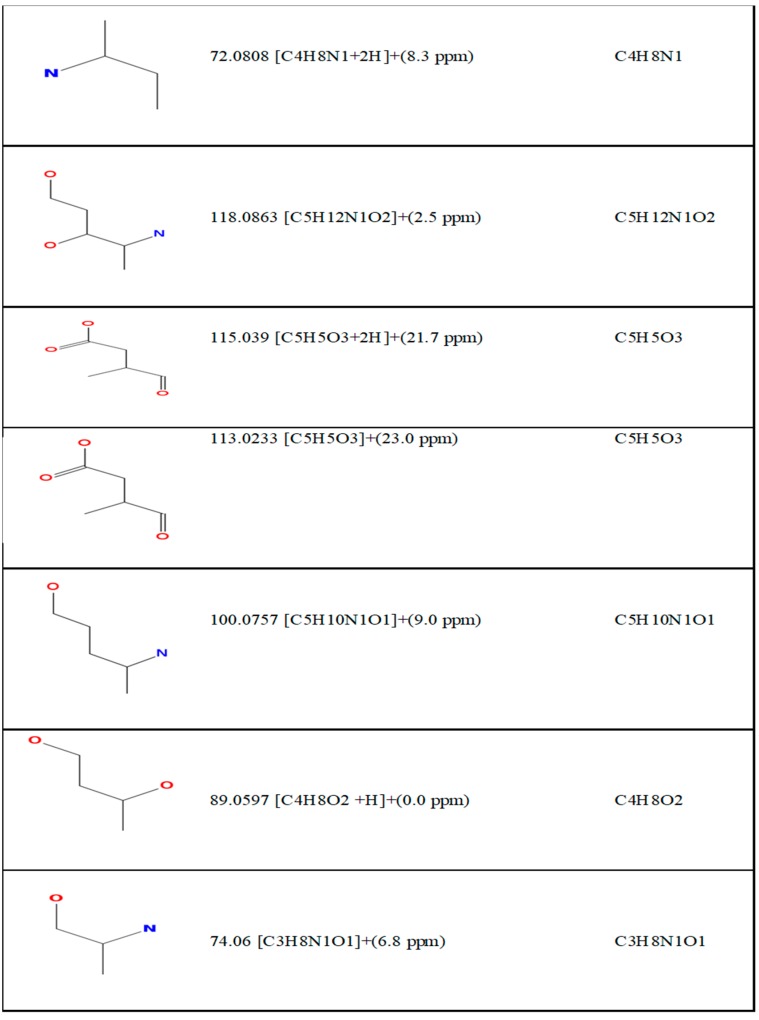
Associated confirmatory MS/MS spectral interpretation and identification of FB_1_ taking into fragments identified using database searches via MetFrag.

It was possible to further confirm the identities of analytes based on exact masses and corresponding MS spectral outputs and searches on various database software. MS/MS spectra allowed for interpretation of compound spectra based on fragmentation patterns and integrated scores. MassBank, MetFrag, and MetFusion software were in this instance used. An example of compound spectra used for FB_1_ is shown in Figure 4 with associated confirmatory MS/MS spectral interpretation and identification (MetFrag) depicted in Figure A2.

The general contamination profile obtained from analysis of these dairy cattle feeds revealed a 40/40 frequency rate of aflatoxin G_2_ (AFG_2_), and derivatives α-zearalenol (α-ZEL) and β-zearalenol (β-ZEL). Aflatoxin B_2_ (AFB_2_) was present in 37/40 of samples while beauvericin (BEA) was found in 36/40 of all samples. HT-2 toxin (HT-2) and fumonisin B_1_ (FB_1_) showed 35/40 and 34/40 frequency rates respectively. Deoxynivalenol (DON) and zearalenone (ZEN) were positive in 24/40 of all samples while aflatoxins AFG_1_ and AFB_1_ showed the least contamination rates at 22/40 and 19/40 respectively. Citrinin (CIT) and ochratoxin A (OTA) were not detected in all 40 tested samples.

### 2.3. Quantitative Screening

#### 2.3.1. Method Performance Characteristics

The limits of detection (LOD) and limits of quantification (LOQ) were determined from chromatograms of the lowest analyte concentration based on respective signal-to-noise (S/N) ratios of 3:1 and 10:1, respectively. Limits of detection ranged from 0.02 (AFB_1_) to 3.46 ppb (FB_1_), while limits of quantification ranged from 0.06 (AFB_1_) to 11.52 ppb (FB_1_). Sensitive MS detection enhanced by the pre-concentration steps in sample preparation allowed for the low LOD and LOQ values obtained. Overall, 14 of the studied 15 mycotoxins were quantified from various dairy feedstuff with an average of 9 different metabolites detected per sample. Overall data on occurrence, concentration ranges, means, and method limits per standard are compiled in Table 1.

Apparent recoveries were based on four spiked model blank matrices and the results are presented in Table 2 (only analytes with reference material available for quantification purposes are shown). For AFG_1_, the highest to lowest apparent recovery percentages were found in bran (76 ± 4), maize (73 ± 12), brewers spent grains (61 ± 18), and maize silage (54 ± 8). Subsequent quantification results were not corrected for apparent recoveries due to the wide nature of sample matrices and inadequate data.

Quantification was performed based on external calibration using serial dilutions of multi-analyte standard stock solutions in solvent. Linear calibration curves of the type *y* = *mx* + *c* with no weighting and an average of 5 data points were used. Satisfactory coefficients of determination (R2) of greater than 0.998 were found for all analytes indicating satisfactory linearity across concentration ranges used.

#### 2.3.2. Natural Occurrence of Mycotoxins in Dairy Feed Samples

**Figure 5:**
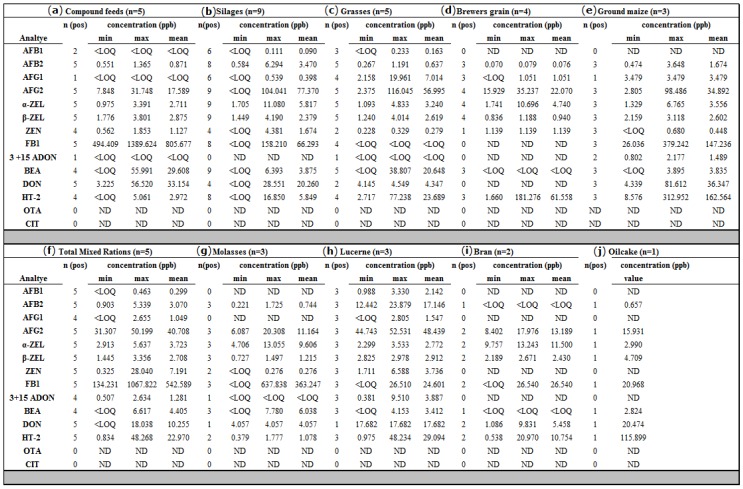
Summary statistics for the mycotoxins detected and quantified in samples of (**a**) compound feeds, (**b**) silages, (**c**) grasses, (**d**) brewers spent grains, (**e**) maize feeds, (**f**) total mixed rations, (**g**) molasses, (**h**) lucerne (**i**) bran and (**j**) oilcake. [AFB_1_ (Aflatoxin B_1_), AFB_2_ (Aflatoxin B_2_), AFG_1_ (Aflatoxin G_1_), AFG_2_ (Aflatoxin G_2_), α-ZEL (Alpha-zearalenol), β-ZEL (Beta-zearalenol), ZEN (Zearalenone), FB_1_ (Fumonisin B_1_), BEA (Beauvericin), DON (Deoxynivalenol), HT-2 (HT-2 toxin), OTA (Ochratoxin A), CIT (Citrinin), and 3 + 15 ADON (3-acetyldeoxynivalenol and 15-acetyldeoxynivalenol)]. Calculation of mean and range values was based on positive samples. ***n*** (**pos**): number of positive samples.

Forty randomly selected dairy feed samples from different classes of ingredients were analyzed for AFB_1_, AFB_2_, AFG_1_, AFG_2_, OTA, CIT, ZEN, α-ZEL, β-ZEL, DON, HT-2, BEA, FB_1_, and 3- & 15-acetyldeoxynivalenols (ADONs). A descriptive statistical analysis of the data on the natural occurrence of mycotoxins per feedstuff category assessed is presented in Figure 5. Findings demonstrate that various feed groups including commercial compound feeds, maize, and silages were contaminated by multiple mycotoxins.

Concerning the AFs, AFB_1_, and AFB_2_ were found in 47.5 and 92.5% of the samples, respectively, while AFG_1_ and AFG_2_ had respective incidence rates of 55 and 100%. However, AFG_2_ and AFB_2_ were the most frequently found AFs. Overall, AFG_2_ had the highest concentrations detected at levels exceeding the FDA legislated action levels for total aflatoxins in dairy feeds (20 ppb). These were detected in silages (mean level: 77.4 ppb; range: <LOQ—104.1 ppb), grasses (mean level: 56.9 ppb; range: 2.4–116.1 ppb), lucerne based feeds (mean level: 48.4 ppb; range: 44.7–52.5 ppb), total mixed rations (mean level: 40.7 ppb; range: 31.3–50.2 ppb), ground maize (mean level: 34.9 ppb; range: 2.8–98.5 ppb), and brewers spent grains (mean level: 22.1 ppb; range: 15.9–35.2 ppb). The highest AFG_2_ levels were found in a sample of dried grass (116.1 ppb) and maize silage (90.7 ppb), all from one farm plus two other independently sourced samples of maize silage (104.1 ppb) and maize grain (98.5 ppb). At 92.5% incidence, AFB_2_ was highest in lucerne samples with a mean level of 17.1 ppb (range: 12.4–23.9 ppb) recovered. AFG_1_ was detected at the highest amounts in grasses (mean level: 7.0 ppb; range: 2.2–19.96 ppb).

AFB_1_ detected levels were highest in lucerne based feeds at mean levels of 2.1 ppb (range: 0.98–3.33 ppb). No AFB_1_ was detected in all samples of molasses meal, brewers spent grain, bran, maize, and oilcake analyzed.

Fumonisin B_1_ contamination in feeds tested displayed an incidence of 85%, with 22/34 (64.7%) of the positive samples being quantifiable (>LOQ) at a mean level of 373.0 ppb (range: <LOQ—1389.6 ppb). Highest levels of FB_1_ were found in compound feeds (mean level: 805.7 ppb; range: 494.4–1389.6 ppb), total mixed rations (mean level: 542.6 ppb; range: 134.2–1067.8 ppb) and molasses (mean level: 363.2 ppb; range: <LOQ—637.8 ppb), while the lowest amounts were found in lucerne (mean level: 24.6 ppb) and oilcake (mean level: 21.0 ppb). FB_1_ in grasses was below the limit of quantification while it was not detected at all in brewers spent grains. Within the groups of compound feeds and mixed rations, substantial amounts of FB_1_ were found in one sample of commercial dairy pellets (1389.6 ppb) and two independently sourced total mixed ration samples (1067.8 and 890.9 ppb, respectively). Regarding the trichothecenes (THs), HT-2 had the highest incidence and level of contamination (87%, mean level: 35.1 ppb, range <LOQ—312.9 ppb), followed by DON (60%, mean level: 20.4 ppb, range: <LOQ—81.6 ppb) and its acylated derivatives 3 and 15-ADONs. The 3- and 15-acetyl deoxynivalenol (ADONs- in combination) were found in trace amounts in 30% of all samples (mean level: 2.2 ppb; range: <LOQ—9.5 ppb). High amounts of HT-2 were detected in 2/3 of the maize samples with ground maize at 312.9 ppb and maize grits at 166.2 ppb plus individual samples of brewers spent grain (181.3 ppb), oilcake (115.9 ppb), and dried grass (77.2 ppb). Highest levels of DON were detected in individual samples of independently sourced maize (81.6 ppb), dairy maize meal (56.5 ppb), and dairy pellets (51.9 ppb), of which the last two were commercially produced.

Low concentrations of ZEN were found in 60% of the tested samples (mean level: 2.8 ppb; range: <LOQ—28.0 ppb). Although it was not detected in bran and oilcake samples, the total mixed rations appeared to be the most contaminated group due to what seems to be an outlier sample of commercial semi-complete total mixed ratio (28.0 ppb). All other samples appeared to have reasonably low levels of ZEN (range: <LOD—6.6 ppb). Zearalenone metabolites, α-ZEL and ß-ZEL were detected in the feeds at a 100% incidence in trace amounts, although the more estrogenic α-ZEL was detected at higher levels (mean level: 4.8 ppb; range: 0.9–13.2 ppb) than the less estrogenic ß-ZEL (mean level: 2.4 ppb; range: 0.7–4.7 ppb).

Beauvericin was found at a high 90% incidence with 18/36 of the positive samples quantifiable (mean level: 8.8 ppb; range: <LOQ—56.0 ppb). Apart from slightly elevated levels in two independent samples of dairy pellets (56.0 ppb) and dried grass (38.8 ppb), all other BEA contaminations were at trace levels (<LOQ—7.8 ppb). Ochratoxin A (OTA) and citrinin (CIT) were not detected in the analyzed samples in this study.

## 3. Discussion

The persistent occurrence of mycotoxins in both food and feeds constitutes a basic challenge to food safety. Since mycotoxin contamination in diets for dairy cattle can be carried over to humans via consumption of by-products from animals fed mycotoxin contaminated feed [5], understanding farmers’ perception toward the mycotoxin problem is key to finding working solutions for mycotoxin management. While this study revealed that there is general knowledge on mycotoxins, there were still several gaps on related topics such as causes of mycotoxins, prevention, health implications, and control. Moreover, there is a low adoption of common mycotoxin control strategies available to these commercial dairy farmers. Even where used, farmers remained generally unaware of the extent to which these adoptions could limit mycotoxins in feeds.

Detection and quantification of mycotoxins were achieved using the robust and sensitive UHPLC-QTOF-MS/MS. A simple and rapid method for determining small molecules described by the instrument manufacturer (Bruker Daltonics, Germany) was adapted and used with slight modification, for maximum sensitivity of mycotoxins. Formic acid was added to the mobile phase to act as an ionization agent and subsequently improve peak shape and MS signals of some analytes. Methanol-formic acid aqueous and acetonitrile-formic acid aqueous solutions were evaluated as mobile phases and the methanol-formic acid aqueous combination was subsequently employed as it was found to give better chromatographic results regarding signal sensitivity and peak areas. This was possibly due to methanol’s ability over acetonitrile to effectively protonate some mycotoxins, resulting in greater signal enhancement when run in ESI+ mode [12]. The two flow rates investigated (0.4 mL/min and 0.3 mL/min) proved the latter to effectively enhance signal sensitivity and decrease run time. This may be because increased flow rates (>0.3 mL/min) tend to increase droplet size while decreasing yield of charged droplets from ESI [12]. Satisfactory sensitivity for target compounds and MS data was obtained by applying the ESI interphase in positive auto MS/MS acquisition mode.

### 3.1. Natural Occurrence in Feeds

Qualitative results (Figure A1) of the analyzed mycotoxins and metabolites in dairy feedstuff obtained from dairy farms in the Gauteng province were somewhat consistent with those of [8], which suggested that AFs, FUMs, DON, and ZEN are common contaminants of South African compound feeds although they reported an absence of HT-2 and a presence of OTA (30%). Quantitative results (Figure 5) on mycotoxin incidence and contamination levels across all 40 samples assessed demonstrated lower level contamination by several mycotoxins, although several feed groups (commercial compound feeds, maize, silages, and grasses) were shown to be contaminated by multiple mycotoxins, making co-occurrence a rule.

#### 3.1.1. Aflatoxins

These naturally occurring *Aspergillus* derived polyketides are potent liver carcinogens, immunosuppressants, and mutagens capable of causing grave harm across several animal species. Primarily found in a wide range of agricultural produce under favorable conditions at both pre-harvest and post-harvest, animal feeds are a common substrate for AFB_1_, AFB_2_, AFG_1_, and AFG_2_. Results in this study, however, reveal a clear indication of an uncommon AF occurrence pattern with higher amounts of the less potent AFB_2_ and AFG_2_ dihydro-derivatives and lower incidences of AFB_1_ and AFG_1._ Similar findings with high AFG_2_ content and trace amounts of other aflatoxins have been reported in nut and dried fruit samples [13], animal feeds [14], cereal samples [15], raw maize and groundnut samples [16], and palm kernel cake [17]. Mngadi et al. [3], also mentioned similar ratios in preliminary work done on South African animal feeds. Such variations may be attributed to the complex differences in the aetiology in the production of the toxins with a generally accepted understanding that AFB_1_ and AFB_2_ are produced by fungal species belonging to the *A. flavus* while *A. paraciticus* produces all the principal four AFs (AFB_1_, AFB_2_, AFG_1_ and AFG_2_) [18,19,20,21]. Moreover, experimental studies on the issue indicate that AFB and AFG ratios are largely influenced by conditions within ecological niches that parent fungal species occur [16]. Studies by Schmidt-Heydt et al. [22], showed temperature as the key parameter for the proliferation of AFB_1_, whereas a_w_ is vital for AFG_1_ biosynthesis. Lin et al. [23], however, bases B and G ratio on temperature cycling. Holmes et al. [24], uses gene cluster and population analysis to suggests the expression of two environmentally affected AFG producing *A. parasiticus* strains (a high B to G strain vs. a high G to B strain), which reveal a history of mutation favoring the high G to B strain [25]. While there is hardly much occurrence data in this regard, it may hence imply location dependent AFB and AFG concentrations that may evolve due to climate change dynamics.

Due to the excessively high concentrations of AFG_2_, 62.5% (25/40) of all samples tested exceed the 20 ppb FDA regulated limit for total AFs in dairy feeds with all maize silages, lucerne and total mixed ration samples being grossly contaminated and exceeding limits. Amongst the analyzed feeds, those feeds deemed safe for consumption by dairy cattle included some samples of oilcake (*n* = 1), maize (*n* = 2), grasses (*n* = 1), bran (*n* = 2), grass silage (*n* = 1), molasses (*n* = 2), and compound feeds (*n* = 3).

#### 3.1.2. Fumonisins

Fumonisins have been shown to be natural contaminants of maize and its by-products in several parts of the world (Canada, USA, Middle East, Africa, and Europe) [26,27]. The *Fusarium*-derived FB_1_ is toxicologically the second most relevant mycotoxin after AFB_1_ [28,29] and accounts for approximately 70% of total FB (FB_1_ + FB_2_ + FB_3_) content in naturally contaminated agricultural produce [30].

The FB_1_ content in the feedstuff here investigated was however demonstrated to be well within the 20 ppm EC legislated limit for FB_1_ + FB_2_ combination in dairy feeds [31]. Results obtained are comparable with those of [32] where 42 maize samples from South Africa were reported to contain FB_1_, FB_2_, and FB_3_ with FB_1_ at 100% incidence and a maximum concentration 1600 ± 1.3 ppb. Chilaka et al. [33], also gives a similar report for South African maize samples with 100% FB incidence (*n* = 40) and contamination levels in the range 64–1035 ppb. Additionally, Njobeh et al. [8], postulates that surveys on FB levels in South African feeds demonstrated contamination levels within a maximum limit of 6000 ppb as exemplified by [3], at 5900 ± 40 ppb; [27], at 4398 ppb and [8], at 2999 ppb.

The absence of FB_1_ in brewers spent grains concurs with work by [34,35]. According to Kovalsky et al. [36], higher FB_1_ amounts detected in commercial processed or semi-processed samples can be, attributed to the high stability of fumonisins, which renders them readily detectable in processed feeds than the unprocessed compact matrices.

Quantification of FB_2_ was not successful due to the inadequate availability of reference material, however, preliminary work allowed for the identification of FB_2_ from chromatographs and compound spectra in all samples under investigation.

#### 3.1.3. Trichothecenes

These *Fusarium*-derived small molecule toxins of high potency are largely associated with *Fusarium* head blight (FHB) in cereal grains and thus have significant health and economic implications. Of significance to this study was the type A TH, HT-2 as well as the most commonly occurring type B TH, DON alongside its acylated derivatives 3- and 15-ADONs. While the quantified HT-2 levels were within the recently placed EC recommended level of 500 ppb for cereals meant for animal feeds, inadequate sub-Saharan Africa data for HT-2 contamination, let alone South Africa leaves little or no room for comparison in terms of occurrence. Existing data indicate 1–8% incidence rates in Nigerian cereals [37], 25% (*n* = 60) in Tanzanian maize (range: 15–25 ppb) [38] and no positive published data for South Africa as zero detection of the toxin has been reported thus far [8,36]. This is thus the first report on HT-2 in South African feeds as HT-2 was previously considered a problem in colder European climates.

Significant DON contamination of feeds has been reported worldwide, as identified in published reviews [39,40,41,42], however, there is still limited information for South African feeds (although much has been done on foods for human consumption). Rodrigues et al. [27], reports an 82% DON incidence in South African feedstuff at mean levels of 943 ppb and maximum 11,022 ppb. Njobeh et al. [8], found DON in 96% South African cattle feeds with a mean of 891 ± 512 ppb and maximum level of 2280 ppb while [36] reported low yearly DON median concentrations (2012–2015) in the range 50–300 ppb for feedstuff investigated. Results of this study, however, showed considerably lower levels of DON (maximum level 81.6 ppb) than reported in the past. Occurrence levels remained well within the EC maximum recommended limit of 2000 ppb for dairy feeds [31]. Of interest, however is specific data on South African maize samples, where Shephard et al. [43], reported on a comparison study of good grain and moldy grain in which respective DON mean levels of 4.7 ± 2.1 ppb and 5.8 ± 2.6 ppb were found, while [32] detected no DON in 42 maize samples tested.

The conjugated derivatives of DON, 3- and 15-acetyl deoxynivalenol were dealt with in combination (ADONs) due to their isometric and co-eluting nature. The lower incidence and levels of the derivatives detected could be attributed to the fact that while stable in solid form, the conjugated mycotoxins are prone to degrade to the parent mycotoxin in methanol or other aqueous solvents [44], which in our case was largely used as the extraction solvent and/or mobile phases.

#### 3.1.4. Zearalenones

The *Fusarium* produced zearalenone (ZEN) is increasingly being recognized as an animal feed contaminant largely affecting cereal crops and bovine forages [45,46,47]. While it exhibits a low acute toxicity, ZEN and its metabolites strongly interfere with animal reproductive systems, decreasing fertility and/or inducing fibroid adenomas, cancers, and carcinomas [48].

Regarding occurrence in South Africa, Boutigny et al. [49], reported it in 33% of naturally infected field-grown maize samples assessed with an average contamination of 34 ppb and a maximum of 67 ppb. Njobeh et al. [8], also documented a 79% ZEN incidence in cattle feeds at maximum level of 123 ppb (mean 72 ± 4.3 ppb). Hickert et al. [32], reported 17% incidence in maize samples available on the market (mean: 36 ± 2.4 ppb; range: 12 ± 0.6 to 73 ± 0.2 ppb). These lower concentrations reported imply the suitability of these commodities for consumption by dairy cattle as per EC standards, and results on our studied Gauteng farm feeds remain no different with ZEN found in 60% tested samples and quantification attainable in 21 of the 24 positive samples at similarly low levels (mean: 2.8 ppb; range: <LOQ—28.0 ppb). Overall low levels reported indicate that ZEN is perhaps a persistent yet minor contaminant of feedstuff in South Africa. Occurrence of the metabolites of ZEN, isomers, α-ZEL and ß-ZEL have been reported in maize, maize products, maize silage, and soya meals at low levels of contamination [50].

#### 3.1.5. Beauvericin

Listed as one of the emerging mycotoxins, the *Fusarium*-derived mycotoxin has over the past few years gained more attention with much effort being put into understanding its role as a mycotoxin with high incidence in many foods and feeds. The occurrence of BEA in samples investigated in the current study may be interpreted as a low but persistent contamination. Similarly, Ezekiel et al. [51], found 100% BEA incidence in poultry feeds from Nigeria (mean: 15 ppb; range: 3–39 ppb). [43] also found a 100% BEA incidence at low concentrations (mean 19 ppb) in good South African maize samples while moldy samples showed higher levels (mean 238 ppb). While there is no published data on the potential toxicity of BEA in cattle, there is still a risk posed by such low concentrations justified by its known toxicities to a wide range of other mammalian cells, additionally, its co-occurrence represents a serious risk for animal health [52].

#### 3.1.6. Ochratoxin A

Despite OTA contaminating a wide range of products, cereal grains within a wide array of ecological niches remain the primary target of the *Aspergillus* and *Penicillium* derived nephrotoxin [53]. Particularly harmful to monogastrics such as pigs and poultry, OTA is however rapidly degraded by the rumen flora of healthy matured ruminants, hence the toxin poses limited risk to bovine species [54]. Njobeh et al. [8], reported a 4% OTA incidence in 92 South African compound feeds with detectable levels sorely found in cattle feeds (mean: 9.9 ppb; range: 6.4–17.1 ppb). However, in compliance with our results, Mngadi et al. [3] and Shephard et al. [43], reported no OTA contamination in South African feeds and feed ingredients tested. While detected in low quantities in the aforementioned study by [8] these specified datasets may be indicative of the scarcity of reported cases of OTA contamination in South African cattle feeds. The common association of OTA with the nephrotoxic CIT based on similar fungal origins (*Aspergillus* and *Penicillium*) may also explain the absence of CIT in the current study.

### 3.2. Mycotoxin Co-Occurrence

The co-occurrence of several mycotoxins in dairy feeds and their raw materials was observed, with an average of 9 out of the 14 tested mycotoxins per individual sample (Figure A1). Four of the forty analyzed samples were contaminated by 12/14 toxins analyzed with CIT and OTA being the exception. These included two samples of commercially produced dairy pellets (protein supplements) from the same farm plus two other independently sourced samples of commercial semi-complete total mixed ration and lucerne pellets, bringing to question why overall data pointed to processed feeds appearing more contaminated than raw feed products. This may be related to the fact that general cereal processing largely concentrates mycotoxins into fractions that are used as animal feedstuff [55].

The co-occurrence of *Aspergillus* metabolites (AFB_1_, AFB_2_, AFG_1_ and AFG_2_) was detected in 37.5% of all samples, which predominantly were dried grasses, lucerne, maize silage, and total mixed rations. The co-occurrence of regulated mycotoxins (AFs, FB_1_, DON, ZEN and HT-2) was detected in 35% of all samples. These low co-occurrence levels, however, are of significance due to the eminent potential for additive or synergistic effects whose full extent and impact in host animals remain to be wholly understood [42].

Of further interest is the co-existence of AFs and FUMs, which have been postulated to have both additive and/or synergistic effects on the host and hence deemed a potential risk factor for liver cancer in some species [56]. Natural co-occurrence of AFB_1_ and FB_1_ in the current study showed a 45% co-occurrence (*n* = 40) with mean AFB_1_ and FB_1_ contaminations levels being 0.74 and 426.1 ppb, respectively.

Commonly reported co-contaminants of cereal grains and most animal feeds are the *Fusarium* toxins ZEN and DON [46,48]. Regardless of their low contamination levels in this study, the two were still found to co-exist in 42.5% of all samples. Driehuis et al. [57], reports that DON and ZEN appear to be less significant contaminants of grass silages compared to maize silages. This remains in agreement with the results of this study where DON and ZEN were both not found in grass silages, while maize silages had DON detected in 4/8 of the samples (mean 20.3 ppb) and ZEN also in 4/8 of the samples (mean 1.7 ppb) with a 25% DON/ZEN maize silage co-occurrence (*n* = 8). Lower levels of ZEN compared to those of DON corroborates with the findings of [46], which observed similar distribution patterns with higher DON levels.

## 4. Conclusions

This study was conducted with the aim to explore the presence of different mycotoxins found naturally in feeds meant for dairy animal consumption used within the Gauteng province of South Africa, and at the same time assess farmer perceptions and knowledge on the risk of mycotoxins. Despite several published works on multi-mycotoxin contamination within South Africa, little has been done on animal feeds, which one could consider the starting point of human exposure. The special focus on one feed group is in a bid to provide a framework for national interventions to minimize exposure where high levels of contamination are established and as a tool to monitor such interventions where employed. Such interventions could give local farmers the opportunity to fully understand the mycotoxin risk and potential health effects of dietary exposure by spreading nationwide awareness and setting up adequate cattle feed handling/management systems while maintaining adequate routine surveillances in this respect. Additionally, the provision of such data sets may spur up the need to re-address national legislations relating to mycotoxin levels in specific feeds groups as it is beneficial for nations to have their own climate/region specific national policies and limits in place to preserve public health and economies from toxic outcomes.

Current data obtained in this study gives clear evidence of feed contamination by AFs with particular regard to AFG_2_, which was frequent in amounts exceeding the 20 ppb FDA action limit for total AFs in dairy feeds. Although a majority of the other mycotoxins detected showed low individual concentrations, which are well within the EC and FDA action or advisory levels, their co-occurrence poses a great risk. Such co-occurrence constitutes a large potential of associated health effects due to the synergistic and/or additive toxicities of these toxins, which are yet to be fully understood scientifically. As such, there is a need for such studies to provide baseline datasets for further research. The study also showed new and interesting data on the presence of the newly regulated HT-2 toxin at high frequencies within tested the samples, which has not been previously reported in animal feeds from the region. Moreover, results on contamination levels for fungal metabolites α-ZEL, β-ZEL and ADONs, which are scarce in Southern Africa were in this study established.

In conclusion, more efforts need to be geared towards adequate feed safety within the Gauteng province of South Africa and perhaps the country at large in order to ensure passable human and animal health. Using data from this study, it would be worthwhile to create public awareness targeted at key stakeholders on the dangers of mycotoxin contamination in the food and feed chain. Of paramount importance, would be the further education of farmers on the risks associated with mycotoxin contamination in the field and during storage, alongside various implementation strategies to control and minimize the risk of exposure to these contaminants.

## 5. Materials and Methods

### 5.1. Study Sites and Socio-Demographic Survey

**Figure 6:**
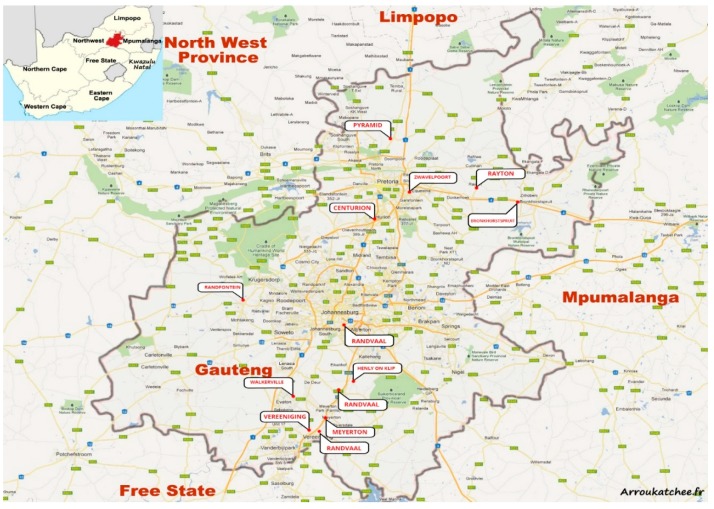
Map of Gauteng Province showing farming areas of interest in this study as identified by name.

The study was undertaken in the Gauteng Province of South Africa, where 13 randomly selected dairy farms from various locations participated (Figure 6). Open- ended and closed-ended self-administered questionnaires were provided to consented farmers representing these farms with the intent to collect information on their socio-demographics, general knowledge, and perceptions on mycotoxins alongside their feed handling and storage habits. This was to statistically access the views and attitudes of the farmers towards mycotoxin contamination and the risk relating them to established mycotoxin contamination levels in their feeds.

### 5.2. Samples

A total of 40 dairy cattle feed samples were during the months between February and April 2015 collected from 13 voluntarily participating farming sites (Figure 6). Samples comprised of maize and grass silages [*n* = 9], grasses (teff, clover, rye, lucerne, chicory, and dried grasses) [*n* = 7], commercial dairy meals/pellets [*n* = 6], total mixed rations [*n* = 5], brewers’ grains [*n* = 4], ground maize [*n* = 3], molasses [*n* = 3], bran [*n* = 2] and oilcake [*n* = 1]. Incremental sampling was performed using samples from various sampling points within the lot. These were thoroughly mixed to give a total of ±700 g per sample and put into sterile, airtight sealed plastic bags. Samples were kept chilled and immediately transported to the Food Technology Laboratories, University of Johannesburg, where they were kept frozen at −4 °C until analysis. Samples were milled (particle size ~10 μm) using an IKA M20 laboratory mill (Merck, Darmstadt, Germany) and homogenous samples were analyzed.

### 5.3. Standard Solutions and Reagents

Mycotoxin standards including of ZEN, α-ZEL, β-ZEL, CIT, BEA, HT-2, and FB_1_ were purchased from Sigma-Aldrich (Steinheim, Germany), while AFB_1_, AFB_2_, AFG_1_, AFG_2_, OTA, DON, 15-ADON and 3-ADON were purchased from Trilogy^®^ (Seattle, WA, USA). Methanol and acetonitrile of LC grade for use in the preparation of mobile phases and as organic solvents plus analytical grade formic acid (purity > 98%) were obtained from Sigma-Aldrich (Steinheim, Germany). Ultrapure water was obtained from a Millipore Milli-Q System (Merck, Johannesburg, South Africa). Disposable PVDF Millex ^®^GV filter units, 0.22 µm (Merck Millipore, Ireland) and Whatman^®^ qualitative grade 1 filter paper purchased from Sigma-Aldrich (Johannesburg, South Africa) were also used.

Two multi-analyte stock solutions were used in the preparation of representative calibration curves and for spiking of blank samples for recoveries. These standard stock solutions constituted:

- ***Aflatoxin mix*:** AFB_1_, AFB_2_, AFG_1_ and AFG_2_ in acetonitrile at concentrations of 50 ppb each.
- ***Multi mix*:** DON, FB_1_, BEA, and CIT at individual concentrations of 2000 ppb each; HT-2, ZEN, α-ZEL, β-ZEL, 15-ADON and 3-ADON at 500 ppb each; and OTA at 135 ppb.

All standard solutions were prepared fortnightly and stored in amber vials at −22 °C according to [58], who reported two-year mycotoxin stability at similar conditions. Ad hoc mixed working solutions were hence freshly prepared from stock solutions throughout the experimental period. Five standard concentrations were prepared to calibrate the instruments as well as to establish external calibration curves.

The following extraction solvents were prepared as needed for immediate use and kept at room temperature: acetonitrile/water/formic acid (79:20:1, *v/v/v*) and methanol/water (80:20, *v/v*). Elution solvents mobile phase A (0.1% formic acid in water) and mobile phase B (0.2% formic acid in methanol) were also used freshly prepared.

### 5.4. Sample Extraction

An extraction protocol developed an optimized by [59] was adapted with some modifications.

Homogenous ground samples (5 g) were individually mixed in 50 mL centrifuge tubes with 60 mL of ACN/ H_2_O/HCOOH (79:20:1, *v/v/v*) and shaken at 600 rpm for 120 min on a mechanical shaker. In the case of molasses and brewers grain samples, a similar protocol was applied except that 80% methanol was used as the extraction solvent according to [60] and [58] who suggested that high sugar content in such matrices could result in layer separation of ACN/H_2_O. Samples in extraction solvents were subsequently centrifuged at 6000 rpm for 6 min (15 °C). Supernatants were collected and filtered through normal grade 1 filter paper to remove larger particles. Precisely 20 mL aliquots of filtered sample extracts were thenceforth evaporated to dryness under a stream of nitrogen gas at 60 °C, followed by reconstitution of the residues with 0.8 mL of ACN/H_2_O (50:50, *v/v*) to ensure preconcentration of all extracts by a factor of 25. Dissolved extracts were vortexed and filtered through 0.22 μm particle size PVDF membrane filter units into LC auto-sampler vials. The method of preconcentration followed was as adapted by [61].

### 5.5. LC-QTOF-MS/MS Conditions

Analysis was performed on an LC-QTOF system equipped with a Dionex UltiMate 3000 UHPLC system (Thermo Scientific, Darmstadt, Germany) and an Impact II Q-TOF mass spectrometer (Bruker Daltonics, Bremen, Germany) coupled to an electro spray ionization (ESI) interphase. Exactly 5 µL of sample extracts were injected into the system with column oven temperature adjusted to 35 °C. The chromatographic separation of analytes was achieved following a gradient elution program (Table 3) on solvent A and solvent B at a flow rate of 0.3 mL/min. ESI-MS/MS was performed in an auto MS/MS mode, which allowed for the automatic selection of three of the most intense precursor ions and subjecting those ions to MS/MS with a cycle time of 0.5 s.

Data acquisition was done in positive ion mode (ESI+). Mass range of the MS scan was set to extend from 50–1600 *m/z*. Interphase parameters included; Capillary voltage of +4.5 kV, dry gas temperature of 220 °C, dry gas flow of 8.0 L/min and nebulizer gas pressure of 1.8 Bar.

External calibration based on serial dilutions of the multi-analyte standard solutions was used for quantification. The linearity was determined by analysis of several increasing concentrations of the standard multi-analyte working solutions in triplicate. Final working concentrations were:

- 2.5, 5, 15, 25 and 50 ppb for AFB_1_, AFB_2_, AFG_1_ and AFG_2_
- 50, 100, 200, 500, 1000 and 2000 ppb for DON, FB_1_, BEA, and CIT
- 12.5, 25, 50, 125, 250 and 500 ppb for HT-2, ZEN, α-ZEL, β-ZEL, 15-ADON and 3-ADON
- 3.375, 6.75, 13.5, 33.75, 67.5 and 135 ppb for OTA.

Linear calibration curves with correlation coefficients of ≥0.997 were hence deduced by plotting signal intensities against concentration of the analyte. Apparent accuracies were determined for external calibrations at 5 concentration levels run in sequential mode and used for further confirmation of calibration accuracy. Limit of detection (LOD) established was at the lowest evaluable concentration levels estimated at a signal-to-noise ratio (S/N) of 3:1, while limit of quantification (LOQ) was similarly estimated at an S/N of 10:1. For instrument control and data acquisition, HyStar™ Version 2.10 was used, while data processing was performed using Compass DataAnalysis 4.3 analytical software and excel worksheets. In addition to the identification of compounds using reference standard materials, confirmatory MS/MS spectral interpretation and identification was done using in silico fragmentation platforms MetFrag and MetFusion to find target candidates.

Recovery experiments were conducted in triplicates on the four least contaminated samples from each subgroup by spiking each of them with multi-analyte solutions of known mycotoxin concentrations. Prior to analysis, spiked samples were kept in the dark overnight to allow equilibration between the samples and the analytes of interest. Subsequent extraction and preconcentration were performed as previously described. Percentage recovery (R) was calculated according to the following Equation (1):

(1) $$R=\frac{Mycotoxin\;concentration\;of\;spiked\;sample-Mcycotoxin\;concenrtration\;of\;unspiked\;sample}{Concentration\;of\;the\;mytoxin\;spiked}\;\times \;100$$

### 5.6. Ethical Clearance of the Study

This study was approved by the Ethics Committee of the Faculty of Sciences, University of Johannesburg (Protocol No: 15042014), approved on 15 April 2014. 

## Figures and Tables

**Table 1:** Mycotoxin levels in feeds from dairy farms in the Gauteng province. Data showing the affiliated method LODs and LOQs per analyte.

Concentration of Positive Feedstuff Samples (ppb) N = 40								
Detected Analyte	*n* (pos)	Incidence (%)	>LOQ (%)	Min (ppb)	Max (ppb)	Mean (ppb)	LOD (ppb)	LOQ (ppb)
AFB_1_	19	47.5	57.9	<LOQ	3.33	0.74	0.02	0.06
AFB_2_	37	92.5	97.3	<LOQ	23.88	3.06	0.05	0.16
AFG_1_	22	55.0	72.7	<LOQ	19.96	2.55	0.05	0.15
AFG_2_	40	100.0	97.5	<LOQ	116.04	41.27	0.06	0.19
α-ZEL	40	100.0	100.0	0.975	13.24	4.84	0.19	0.63
β-ZEL	40	100.0	100.0	0.727	4.71	2.40	0.19	0.64
ZEN	24	60.0	87.5	<LOQ	28.04	2.84	0.04	0.12
FB_1_	34	85.0	64.7	<LOQ	1389.62	372.96	3.46	11.52
ADONs	12	30.0	75.0	<LOQ	9.51	2.20	0.08	0.27
BEA	36	90.0	50.0	<LOQ	55.99	8.81	0.66	2.19
DON	24	60.0	83.3	<LOQ	81.61	20.40	0.49	1.62
HT-2	35	87.5	94.3	<LOQ	312.95	35.11	0.06	0.21
OTA	0	0	ND	ND	ND	ND	0.08	0.26
CIT	0	0	ND	ND	ND	ND	0.13	0.42

***n*** **pos**: number positive samples (number of samples above LOD); **%** (>**LOQ**): percentage of quantifiable positive samples (percentage above LOQ); **LOD**: limit of detection; **LOQ**: limit of quantification; **ND**: not detected. **AFB_1_** (Aflatoxin B_1_), **AFB_2_** (Aflatoxin B_2_), **AFG_1_** (Aflatoxin G_1_), **AFG_2_** (Aflatoxin G_2_), **α-ZEL** (α-zearalenol), **β-ZEL** (β-zearalenol), **ZEN** (Zearalenone), **FB_1_** (Fumonisin B_1_), **ADONs** (Acetyldeoxynivalenols), **BEA** (Beauvericin), **DON** (Deoxynivalenol), **HT-2** (HT-2 toxin), **OTA** (Ochratoxin A), **CIT** (Citrinin).

**Table 2:** Apparent recoveries of studied mycotoxins in four different model matrices.

Mycotoxin	Apparent Recoveries in Model Matrices (%)			
	*Maize*	*Maize Silage*	*Brewers Spent Grain*	*Bran*
Aflatoxin B_1_	177 ± 9	114 ± 8	-	-
Aflatoxin B_2_	71 ± 8	84 ± 12	-	-
Aflatoxin G_1_	73 ± 12	54 ± 8	61 ± 18	76 ± 4
Aflatoxin G_2_	48 ± 13	49 ± 11	44 ± 12	49 ± 9
Acetyldeoxynivalenols	-	-	-	174 ± 6
α-zearalenol	100 ± 5	99 ± 4	99 ± 12	100 ± 7
β-zearalenol	100 ± 9	99 ± 9	102 ± 5	99 ± 5
Citrinin	26 ± 8	-	129 ± 13	-
Beauvericin	98 ± 12	66 ± 5	16 ± 4	48 ± 16
Deoxynivalenol	103 ± 12	105 ± 12	-	-
HT-2 toxin	41 ± 7	-	-	24 ± 6
Ochratoxin A	71 ± 21	37 ± 13	187 ± 9	63 ± 12
Zearalenone	151 ± 12	174 ± 8	-	153 ± 11

**Table 3:** Gradient program for LC.

Time (min)	Solvent A (%)	Solvent B (%)
0	98	2
1.0	98	2
8.0	0	100
12.0	0	100
12.1	98	2
14.0	98	2

**Figure A1:** Qualitative results indicative of co-occurrence of mycotoxins in all feeds tested. Color coded cells represent positives. No Ochratoxin A and Citrinin were detected across all samples.

**Table:** 

Sample	Sample Type	REGULATED MYCOTOXINS								OTHER MYCOTOXINS			
		AFB_1_	AFB_2_	AFG_1_	AFG_2_	ZEN	FB_1_	DON	HT-2	BEA	ADONs	α-ZEL	β-ZEL
# 12	Pellets												
# 29	Dairy maize meal												
# 33	Dairy maize meal												
# 34	Pellets												
# 39	Dairy Pellets												
# 01	Mixed TMR												
# 10	17% TMR												
# 11	19% TMR												
# 23	TMR Semi-complete												
# 40	TMR												
# 26	Oilcake												
# 09	Ground maize												
# 16	Maize grain												
# 28	Maize												
# 02	Teff												
# 06	Dried grass												
# 13	Dried grass												
#14	Dried grass												
# 22	Dried grass												
# 27	Lucerne												
# 37	Lucerne												
# 38	Lucerne Pellets												
# 25	Bran												
# 31	Bran												
#03	Maize silage												
#04	Maize silage												
#07	Maize silage												
#18	Maize silage												
#19	Grass silage												
#21	Silage												
#24	Maize silage												
#35	Silage												
#17	M. silage + BG												
# 08	Brewers grain												
# 20	Brewers grain												
# 32	Brewers grain												
# 36	Brewers grain												
#05	Molasses meal												
#15	Molasses meal												
#30	Molasses												
	POSITIVE* n* pos(N)	19/40	37/40	22/40	40/40	24/40	34/40	24/40	35/40	36/40	12/40	40/40	40/40
